# Estimation of Population-Specific Genetic Parameters Important for Long-Term Optimum Contribution Selection—Case Study on a Dairy Istrian Sheep Breed

**DOI:** 10.3390/ani11082356

**Published:** 2021-08-09

**Authors:** Ante Kasap, Jelena Ramljak, Marija Špehar

**Affiliations:** 1 Faculty of Agriculture, University of Zagreb, Svetošimunska 25, 10000 Zagreb, Croatia; jramljak@agr.hr; 2 Croatian Agency for Agriculture and Food, Svetošimunska 25, 10000 Zagreb, Croatia; marija.spehar@hapih.hr

**Keywords:** inbreeding, effective population size, connectedness, sheep, selection

## Abstract

**Simple Summary:**

Selection progress with minimal loss of genetic variability is a challenging task in small populations exposed to selection, such as the Istrian sheep breed. To achieve a balance between selection gain and loss of genetic variability, genomic optimum contribution selection (OCS) is emerging as the best long-term selection approach. However, investment in genomic OCS requires a deep knowledge of some specific population parameters such as effective population size ($N_{e}$) and connectedness between flocks. The determined $\Delta N_{e}$ suggests recent loss of genetic variability, and low connectedness between flocks makes it difficult to rank animals (breeding values) from different flocks in an unbiased way. The former “calls” for the implementation of OCS to reduce the loss of genetic variability, and the latter for improvement of connectedness in order to conduct a fair genetic evaluation of animals belonging to different flocks.

**Abstract:**

The Istrian sheep breed has been subjected to selection for dairy traits for more than two decades. However, a detailed study of some important population-specific parameters such as effective population size ($N_{e}$) and connectedness between flocks has never been carried out. The aim of the study was to examine the above parameters in dairy Istrian sheep subjected to a national selection program. The $N_{e}$ was estimated as the mean rate of increase in coancestry, and connectedness was determined using four different statistics. The $N_{e}$ was estimated at 73 animals with pedigree constraints imposed on 4 equivalent generations and 3 full generations. Analysis of $\Delta N_{e}$ (“sliding window approach”) revealed a negative $\Delta N_{e}$ indicating a progressive loss of genetic variability ($\Delta N_{e}{}_{\mathrm{NEG}\geq 4}$ = −6.6, *p* < 0.01; $\Delta N_{e}{}_{\mathrm{NFG}\geq 3}$ = −4.9, *p* > 0.05). The overall connectedness ($\bar{r}$ ~ 0.0001) was below the acceptable level for unbiased ranking of the animals belonging to different flocks ($r_{i,j}\;$ = 0.05). OCS appears to be the best option for the long-term survival (self-sufficiency) of the breed, but genetic links between flocks need to be strengthened to allow unbiased ranking of the animals based on the estimated breeding values.

## 1. Introduction

The Istrian sheep is a Croatian dual-purpose breed (milk and meat) with great traditional, cultural, and social importance for the residents of the Istrian peninsula. In recent times, great efforts have been made to keep the breed sustainable, mainly by increasing productivity and economic self-sufficiency through better management and selection. There is a constant market demand for Istrian sheep cheese, which has directed recent selection strategies in the breed towards dairy traits. Performance recording started two decades ago, while BLUP genetic evaluation has been carried out for about a decade [1]. A total of 1632 individuals have been included in the national selection program [2]. For this purpose, the test-day repeatability animal model has been used [3]. For the sake of higher accuracy, the existing genetic evaluation is planned to be upgraded to the single-step genomic BLUP [4]. Increased accuracy of genomic evaluation compared to traditional pedigree-based genetic evaluation was reported in numerous dairy-orientated sheep breeding programs (e.g., [5,6,7,8]).

The small size of the breed requires special attention in making selection decisions due to inevitable inbreeding. Inbreeding is impossible to avoid in small animal populations, especially those under severe selection pressure [9]. The main reason is the overuse of genetically superior, but also genetically more similar, animals. Related animals share genes, so their performance and estimated breeding values are more similar than those of unrelated animals [10]. To control inbreeding, future matings should have a low expected inbreeding coefficient of the offspring, which is equal to the kinship of the parents [11]. The optimum contribution selection approach appears to be the optimal selection strategy in this population to achieve genetic gain and mitigate the loss of selection variability. 

Individual- and population-specific parameters have traditionally been estimated from the pedigree and more recently with genomic data. However, despite the availability of genomic tools, genealogical data still remain the main source of information for monitoring genetic variability in many populations of livestock species [12]. An important measure for monitoring genetic diversity is effective population size (*Ne*), which is the size of an idealized population that would produce the same genetic variation as the population under study [13]. *Ne* can be estimated using a variety of statistics [14]. The most commonly used approach has been the regression of coefficient of inbreeding or coancestry (IBD) on time or generation, with *Ne* = 1/2Δ*IBD*s [15]. One of the more recent approaches estimates individual IBD rates by combining individual IBD coefficients with total equivalent complete generations. This method was originally developed by Gutiérrez et al. [16] and improved by Cervantes et al. [17]. 

The success of across-flock genetic evaluation system depends on the genetic connectedness between flocks. Connectedness indirectly measures the extent to which estimated breeding values can be fairly compared across flocks [18,19]. When flocks are sufficiently connected, the BLUP genetic evaluation is robust, and estimated breeding values (EBV) can be fairly compared between flocks. On the other hand, limited connectedness leads to bias when comparing EBVs of animals belonging to different flocks [20]. Several statistical measures have been developed and proposed so far to examine the degree of connectedness. Some of the most know are connectedness index [21], coefficient of determination of the difference between EBVs of a pair of animals [22], prediction error variance of differences in EBVs between animals, variance of estimates of management-unit effects, gene flow method, genetic drift variance [23], and correlation of breeding value prediction errors [24]. These statistics are useful to estimate the risk of comparing EBVs between flocks, as well as to design breeding schemes aimed at effectively linking flocks. Recently, genomic data have been used to assess connectedness [25,26]. A special tool for this purpose (R package “GCA”) driven by either pedigree data or genomic data (SNPs) has recently become available [27]. 

In order to provide basic information essential for designing the selection strategy in the Istrian sheep population, this study aimed to estimate two very important population-specific parameters: effective population size and genetic connectedness between flocks.

## 2. Materials and Methods

### 2.1. Data

Genealogical data of the Istrian sheep breed for this study were provided by the Croatian Ministry of Agriculture. Istrian sheep included in the Croatian national selection program and all their available ancestors were included in the analysis. A total of 6866 sheep belonging to 118 flocks were included in the analysis. Truncation of the data used in the inferential statistical analysis, i.e., the definition of the reference population, was done at several instances based on different criteria (measures of pedigree information). The statistics used to assess the quality of the pedigree and the thresholds used to truncate non-informative animals are explained in detail in the rest of the text.

### 2.2. Statistical Analysis

#### 2.2.1. Pedigree Analysis

All steps of the statistical analysis were conducted in R programming environment (R Core Team) [28]. The package “optiSel” [29] was used for

(1) Quality control of the pedigree (completeness, pedigree completeness index (*PCI*), number of equivalent complete generations (*NEG*), number of fully traced generations (*NFG*), and number of maximum generations traced (*NMG*)),
(2) Estimation of individual- and population-specific parameters (coefficients of inbreeding ($F_{i}$) and kinship ($K_{ij}$), effective population size ($N_{e}$), and generation interval).

**Figure 1:**
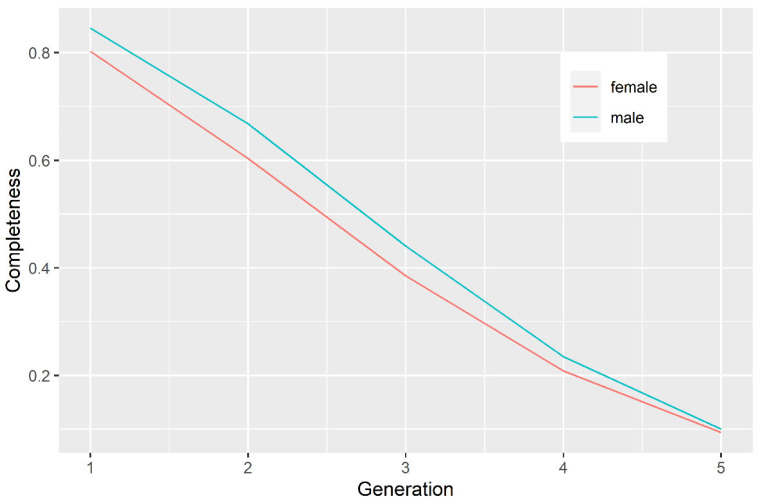
Completeness of the pedigree of Istrian sheep under the study.

**Completeness** was calculated for individuals and groups of individuals in each ancestral generation. This measure represents the proportion of known ancestors in each generation. The results obtained are presented graphically (Figure 1).

**Pedigree completeness index (PCI),** i.e., the harmonic mean of the pedigree completeness of the parents [30], was calculated using the following formula:

(1) $$PCI=\frac{2C_{f}*C_{m}}{2C_{f}\;+C_{m}}$$

with $C_{f}$ and $C_{m}$ being proportions of paternal and maternal ancestors estimated based on:

(2) $$C=\frac{1}{d}\sum_{i=1}^{d}a_{i}$$

where $a_{i}$ was the ratio of known to unknown ancestors in each generation, and d was the number of generations. The harmonic mean ensures that the less complete ancestral pedigree is weighted more heavily, so the *PCI* equals zero when either parent is unknown. Even though in specific occasions inbreeding coefficients can be valid despite small *PCI*s (if the most recent founders were indeed unrelated), speculating with this information would be very risky; therefore, we decided to discard from the analysis all individuals with *PCI* less than 0.6. 

**Equivalent number generations (NEG)** were obtained as the sum of the proportions of known ancestors of an individual over all traced generations [31] as follows:

(3) $$\overset{n_{j}}{\underset{i=1}{\sum }}\frac{1}{2^{g_{ij}}}$$

In the above formula, $n_{j}$ is the number of ancestors of individual *j*, and $g_{ij}$ is the number of generations between individual *j* and its ancestor *i*. In this way, 1/2 is added for each known parent, 1/4 for each known grandparent, 1/8 for each known great-grandparent, and so on. 

**Effective population size (Ne)** was estimated from the mean rate of increase in coancestry [17], where the increase in coancestry between any pair of individuals *i* and *j* was computed as:

(4) $$\Delta C_{ij}=1-\;\sqrt[{\frac{g_{i}+g_{j}}{2}}]{1-C_{ij}}$$

where $C_{ij}$ is the kinship between *i* and *j*, and $g_{i}$ and $g_{j}$ are the numbers of equivalent complete generations of individuals *i* and *j*. The effective size was then estimated as:

(5) $$N_{e}=\frac{1}{2\bar{\Delta C}}$$

The reference population was set to animals born between 2010 and 2018, with the sliding window approach set to a 4-year generation interval. In the inferential analysis pertaining to $N_{e}$, two different scenarios were examined (constraints on *NEG* ≥ 4, i.e., *NFG* ≥ 3). The inbreeding rate (ΔF) and effective population size rate ($\Delta N_{e}$) were estimated by regressing *F* and $N_{e}$ on the generation number.

#### 2.2.2. Connectedness Analysis

All pedigree records were used in the analysis of connectedness, but the attention in this part of the study was paid to 14 flocks that actively participate in the national selection breeding program. These flocks have been subjected to regular milking controls and represent a base for previous and future selection work in this sheep breed. The “GCA” package [27] was used for connectedness analysis. Four genetic connectedness statistics were estimated: 

$PEVD_{ind}$—prediction error variance of differences in EBVs between animals belonging to different flocks [23]. This metric measures the prediction error variance difference of breeding values between individuals from different flocks (management units). This is the most computationally expensive, but most accurate, statistic for this purpose. The prediction error variance (PEV) of the EBVs was obtained from the diagonal elements of the inverse of the coefficient matrix. Using this method, the pairwise PEVDs were first computed at the individual level as follows:

(6) $$PEVD\left({\hat{u}}_{i}-{\hat{u}}_{j}\right)=\left[PEV\left({\hat{u}}_{i}\right)+PEV\left({\hat{u}}_{j}\right)-2PEC\left({\hat{u}}_{i},{\hat{u}}_{j}\right)\right]=\left(C_{ii}^{22}-C_{ij}^{22}-C_{ji}^{22}-C_{jj}^{22}\right)\;*\;\sigma_{e}^{2}$$

and thereafter aggregated and summarized at the unit level as follows:

(7) $$PEVD_{i^{\prime }j^{\prime }}=\frac{1}{n_{i^{\prime }}\;*\;n_{j^{\prime }}}\;\sum PEVD_{i^{\prime }j^{\prime }}$$

$PEVD_{group}$—variance of estimated differences between management units [23]. Using this method, we first calculated the mean prediction error variance of units *i* ($\bar{PEV_{i^{\prime }i^{\prime }}}$) and *j (*$\bar{PEV_{j^{\prime }j^{\prime }}}$) and the prediction error covariance ($\bar{PEC_{i^{\prime }j^{\prime }}}$) between units *i* and *j* and then summarized them as follows:

(8) $$PEVD_{i^{\prime }j^{\prime }\;}=\;\bar{PEV_{i^{\prime }i^{\prime }}}+\bar{PEV_{j^{\prime }j^{\prime }}}+2\bar{PEC_{i^{\prime }j^{\prime }}}$$

**CD**—coefficient of determination of the difference between predicted breeding values [22]. This statistic was obtained by scaling the inverse of the coefficient matrix with corresponding coefficients from the relationship matrix. CD between individuals *i* and *j* was calculated as follows:

(9) $$CD_{ij}=1-\lambda \frac{C_{ii}^{22}+C_{jj}^{22}-2C_{ij}^{22}}{K_{ii}+K_{jj}-2K_{ij}}$$

where $K_{ii}$ and $K_{jj}$ are the *i*th and *j*th diagonal elements of *K*, and $K_{ij}\;$ is the relationship between the *i*th and the *j*th animals. The individual average CD was derived from the average of CD between individuals across two units as follows:

(10) $$CD_{i^{\prime }j^{\prime }}=1-\lambda \;*\;\frac{\frac{1}{n_{i^{\prime }}\;*\;n_{j^{\prime }}}\;*\;\sum (C_{i^{\prime }i^{\prime }}^{22}+C_{j^{\prime }j^{\prime }}^{22}-2C_{i^{\prime }j^{\prime }}^{22})}{\frac{1}{n_{i^{\prime }}\;*\;n_{j^{\prime }}}\;*\;\sum (K_{i^{\prime }i^{\prime }}+K_{j^{\prime }j^{\prime }}-2K_{i^{\prime }j^{\prime }})}=1-\frac{\sum PEVD_{i^{\prime }j^{\prime }}}{\sigma_{u}^{2}\;*\;\sum (K_{i^{\prime }i^{\prime }}+K_{j^{\prime }j^{\prime }}-2K_{i^{\prime }j^{\prime }})}$$

**r**—correlation between predicted breeding values of individuals from different flocks [20] was obtained by transforming the PEV matrix into a prediction error correlation matrix. For animals *i* and *j*, $r_{ij}$ was calculated with the formula:

(11) $$r_{ij}=\frac{PEC\left({\hat{u}}_{i},{\hat{u}}_{j}\right)}{\sqrt{PEV\left({\hat{u}}_{i}\right)*PEV\left({\hat{u}}_{j}\right)}}$$

and thereafter summarized at the unit level as follows:

(12) $$r_{i^{\prime }j^{\prime }}=\frac{\sum PEC_{i^{\prime }j^{\prime }}}{\sqrt{\sum PEV_{i^{\prime }i^{\prime }}*\sum PEV_{j^{\prime }j^{\prime }}}}$$

where $\sum PEC_{i^{\prime }j^{\prime }}$, $\sum PEV_{i^{\prime }i^{\prime }}$, and $\sum PEV_{j^{\prime }j^{\prime }}$ are the sums of the elements $PEC_{ij}$, $PEV_{ii}$, and $PEC_{jj}$, respectively.

## 3. Results

### 3.1. Quality Control of the Pedigree

The descriptive statistics of the pedigree are presented in Table 1. The average *NEG*, *NFG*, *NMG*, and *PCI* were 2.15, 1.46, 3.35, and 0.47, respectively. These low numbers arose from older animals in the pedigree with insufficient number of recorded ancestors. The distribution of *PCI* in classes of size 0.2 was almost uniform, with 40% of the animals in the pedigree with *PCI* above 0.6 (Table 2). The correlation between *PCI* and *NEG* was 0.96, indicating that they assess more or less the same, so the distribution of *NEG* was omitted from results. The distribution of *NFG* showed that ~19% animals had three or more fully traced generations (Table 3).

**Figure 2:**
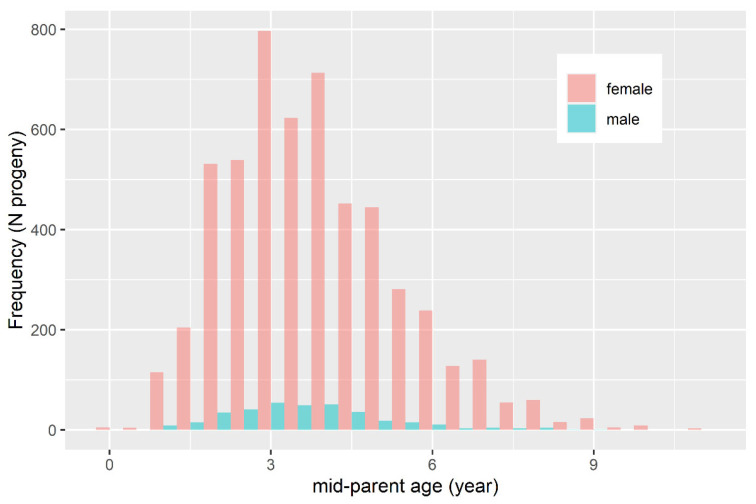
Distribution of mid-parent age of the progeny in the pedigree.

The proportion of known ancestors in each generation (pedigree completeness) is presented on Figure 1. A steep decline in pedigree completeness was observed with each ancestral generation (~20% until generation 4), with males having negligibly more informative pedigree than females. The distribution of the mid-parent age of the animals in the pedigree is presented on Figure 2. Males and females had a similarly shaped distribution. Most animals in the pedigree had a mid-parent age of 3 years, and the generation interval (GI) was estimated to be 3.8 years. 

### 3.2. Reference Population

The reference population was restricted to animals born between 2010 and 2018. The average *PCI* in the reference population was 0.74. The distribution of *PCI* is presented in the Table 4. Of the total of 2226 animals, ~80% had *PCI* > 0.6, and ~60% had *PCI* > 0.8. The distribution of *NFG* for the reference population is presented in Table 5. Approximately one half of the animals (49.46%) had three or more *NFG*. *PCI* and *NFG* were highly correlated (*r* = 0.92), therefore only *PCI* values are reported.

**Figure 3:**
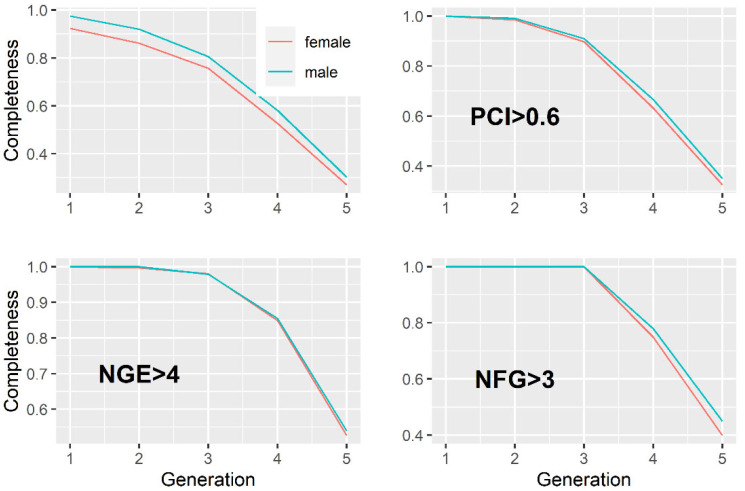
Completeness of the pedigree of the reference population.

The proportion of known ancestors in the reference population for each generation is presented in Figure 3. The completeness of the pedigree of the reference population was improved by imposing constraints on *PCI* ≥ 0.6, *NEG* ≥ 4, and *NFG* ≥ 3.

### 3.3. Individual- and Population-Specific Genetic Parameters

#### 3.3.1. Inbreeding

**Figure 4:**
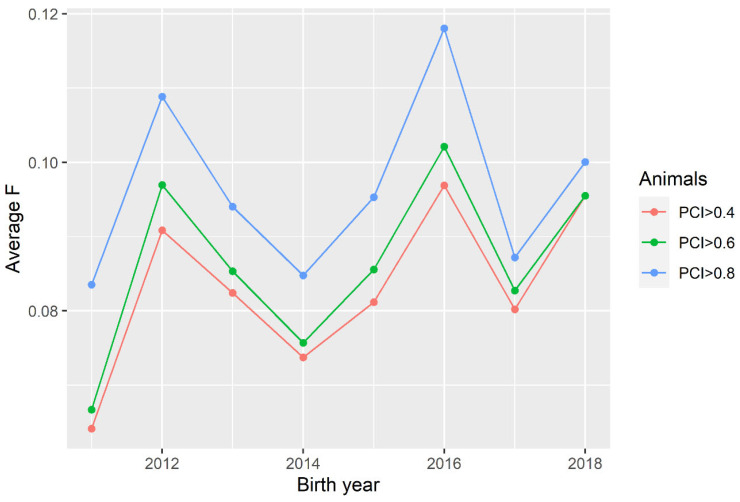
Mean coefficient of inbreeding (*F*) by year of birth for different levels of *PCI*.

The annual inbreeding change in the reference population was consistent across different levels of *PCI* (Figure 4). A consistent change in the magnitude of *F* for different levels of *PCI* nicely reflects how inbreeding in some populations can be underestimated with a distortion of genetic links to common ancestors. A consistent rate of inbreeding across different levels of the examined levels of *PCI* suggests that *Ne* would not be seriously compromised in this population for any level of *PCI* above 0.4. Nevertheless, for the sake of credibility, the additional constraints were imposed on the animals in the reference population for the analysis of *F* and *Ne* (*NEG* ≥ 4 or *NFG* ≥ 3).

The distribution of the coefficient of inbreeding (*F*) in the reference population is presented in Table 6. Three quarters of the animals (~75%) had *F* below 10%. *F* between 11% and 20% was found in 15% of the animals, and an extremely high level of inbreeding (*F* > 20%) was determined in 10% of the population. When constraints were imposed on *NEG* ≥ 4 and *NFG* ≥ 3, the distribution of *F* shifted towards higher values of *F*, with marginal differences between $F_{NEG\geq 4}$ and $F_{NFG\geq 3}$. The average coefficients of inbreeding per generation ($F_{NEG\geq 4}$ and $F_{NFG\geq 3}$) are presented in the last column of Table 7 and Table 8. The estimated inbreeding rates per generation $\Delta F_{NEG\geq 4}$ and $\Delta F_{NFG\geq 3}$ were −0.0013 ± 0.0005 (*p* > 0.05) and 0.003 ± 0.0006 (*p* < 0.05), respectively.

#### 3.3.2. Effective Population Size

Estimates of effective population size ($N_{e}$) for the reference population based on animals with more than four known equivalent generations ($Ne_{NEG\geq 4}$) and more than three known full generations ($Ne_{NFG\geq 3}$) are presented in Table 7 and Table 8. The “sliding window approach” (4-year generation interval) was used in the analysis of the effective population size. The estimated $Ne_{NEG\geq 4}$ ranged from 73 animals in GI = 4 (2015–2018) to 93 animals in GI = 1 (2012–2015). The $Ne_{NFG\geq 3}$ ranged from 73 animals in GI = 3 (2014–2017) to 92 in GI = 1 (2012–2015). The estimated rates per generation ($\Delta Ne_{NEG\geq 4}$ and $\Delta Ne_{NFG\geq 3}$) were similar in magnitude and direction ($\Delta Ne_{NEG\geq 4}$ = −6.6 ± 0.35 (*p* < 0.01) and $\Delta Ne_{NFG\geq 3}$ = −4.9 ± 1.8 (*p* > 0.05)). The estimated ratios $Ne_{NEG\geq 4}/Nt_{NEG\geq 4}$ and $Ne_{NFG\geq 3}/Nt_{NFG\geq 3}$ revealed that $N_{e}$ accounted for 10% to 15% of the census population. An incomplete pedigree from either side (paternal or maternal) leads to overestimation of population-specific parameters such as $N_{e}$, due to the inability to detect recent inbreeding. Therefore, in the absence of recent introgression of foreign genomes (outbreeding), the estimated $N_{e}$ obtained for animals in the pedigree with at least three fully traced generations ($Ne_{NFG\geq 3}$) better reflects true population parameters in this population.

### 3.4. Connectedness

#### 3.4.1. PEVD

In the analysis of connectedness, the original Flock IDs were recoded for the sake of better visibility of results and anonymity of breeders. In addition to these performance tested flocks, several additional flocks were included in the analysis due to their indirect contribution to the formation of genetic links across the pedigree. 

**Figure 5:**
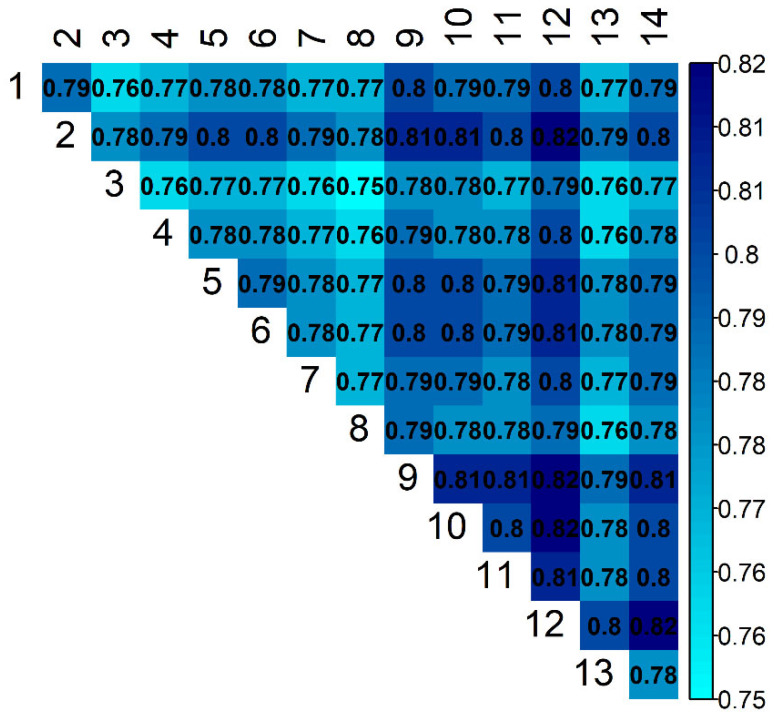
Estimates of connectedness between 14 flocks of Istrian sheep based on $PEVD_{ind\left(i,j\right)}$.

**Figure 6:**
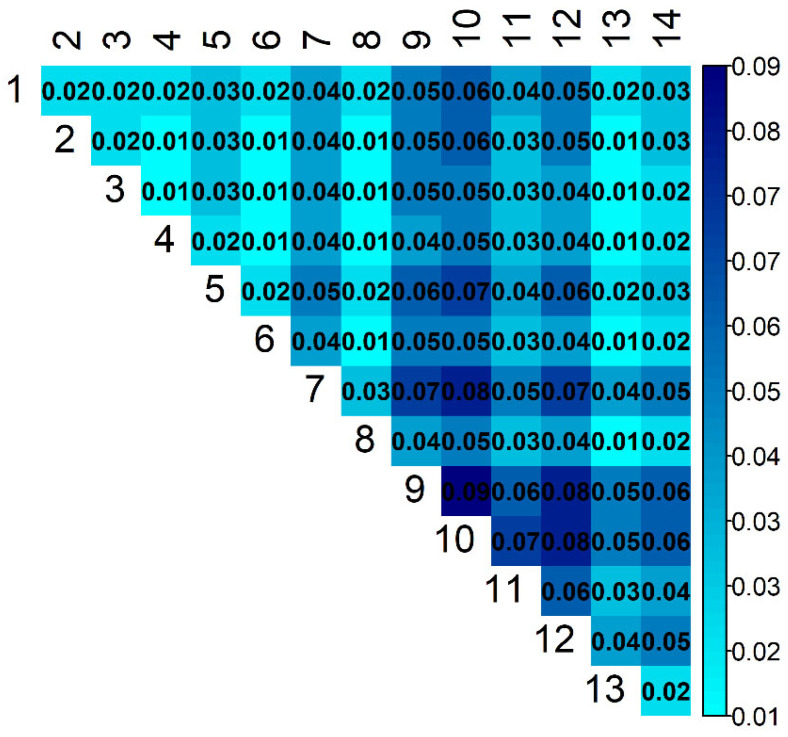
Estimates of connectedness between 14 flocks of Istrian sheep based on $PEVD_{group\left(i,j\right)}$.

Estimates of genetic connectedness obtained with the prediction error variance of the difference ($PEVD_{ind\left(i,j\right)}$ and $PEVD_{group\left(i,j\right)}$) are presented in Figure 5 and Figure 6. The *PEVD* statistic ranges from 0 to 1, with smaller values indicating better connectedness.

**Figure 7:**
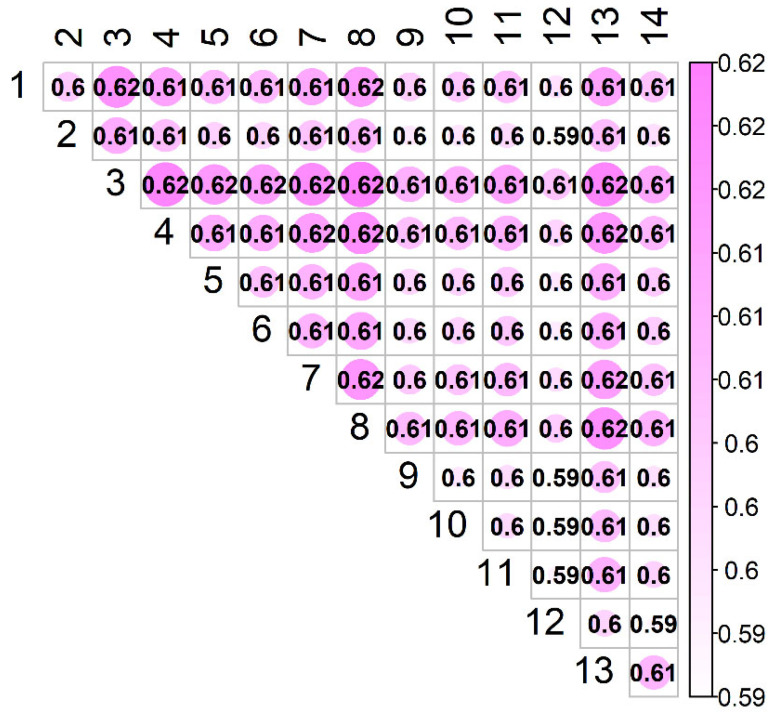
Estimates of connectedness between 14 flocks of Istrian sheep based on $CD_{ind\left(i,j\right)}$.

The estimated pairwise connectedness between flocks ranged from 0.75 to 0.82 ($PEVD_{ind}$) and from 0.01 to 0.09 ($PEVD_{group}$). The overall $\bar{PEVD_{ind}}$ and $\bar{PEVD_{group}}$ were 0.78 and 0.37, respectively. The results obtained from these two *PEVD* methods agreed to some extent, but there were also some discrepancies in the estimates. The correlation between these statistics was 0.69 (Figure 7). Flocks 3 and 8 were mutually the most connected flocks ($PEVD_{ind\left(3,8\right)}$ = 0.75). Flock 3 was the most connected ($\bar{PEVD_{ind\left(3\right)}}$ = 0.76), and flock was 12 the least connected ($\bar{PEVD_{ind\left(12\right)}}$ = 0.75) flock with the rest of the population. 

Although proclaimed to be the most accurate method to estimate connectedness, the results obtained with *PEVD*-derived statistics are difficult to interpret because no benchmark is defined. One can only adhere to the motto “the fewer the better”, but it is not possible to deduce from the results whether the connectedness in this population was sufficient for an unbiased comparison of EBVs of animals belonging to different flocks. 

#### 3.4.2. CD

The estimated coefficient of determination ($CD_{ind\left(i,j\right)}$) between flocks is shown in Figure 7. In contrast to the *PEVD* statistic, the larger the *CD*, the greater the connectedness. As determined by $PEVD_{ind}$, flocks 3 and 8 were the most connected ($CD_{ind\left(3,8\right)}$ = 0.60). In addition, flock 3 was the most connected, and flock 12 was the least connected to the rest of the population ($\bar{CD_{\left(3\right)}}$ = 0.617, ($\bar{CD_{\left(12\right)}}$ = 0.597). The overall $\bar{CD}$ was 0.60. 

**Figure 8:**
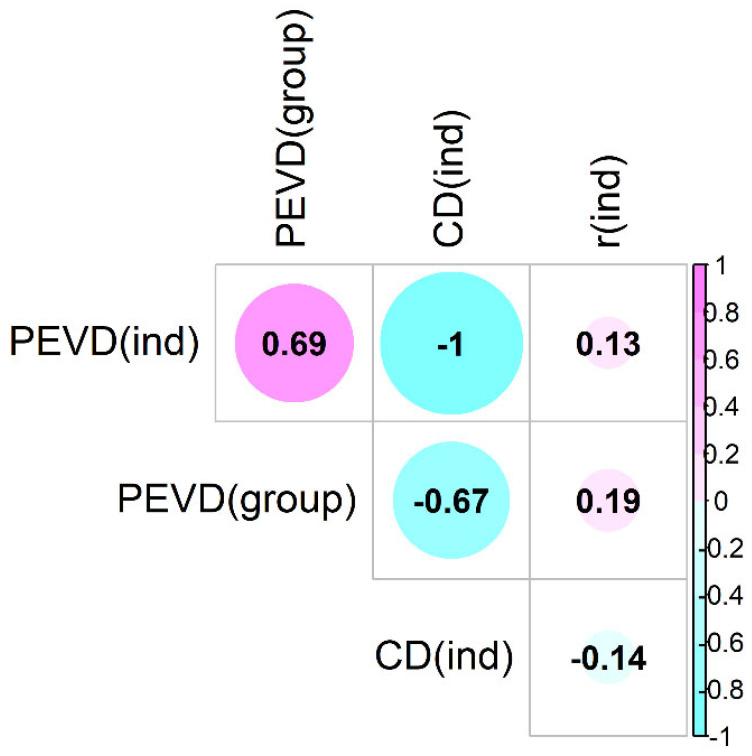
Correlation between four different connectedness statistics.

$CD_{ind\left(i,j\right)}$ was highly correlated to ${\mathrm{PEVD}}_{ind\left(i,j\right)}$ (*r* ~ −1), moderately correlated to $PEVD_{ind\left(i,j\right)}$ (*r* = −0.67), and uncorrelated to $r_{ind\left(i,j\right)}$ (*r* = −0.14) (Figure 8). The benchmark for these statistic ($PEVD_{ind\left(i,j\right)}$) is unknown as well.

#### 3.4.3. r

The estimated coefficients of prediction error correlation ($r_{i,j}$) between flocks were practically 0. The overall $\bar{r}$ was ~ 0.0001. As for the *CD*, the larger the ($r_{i,j}$), the greater the connectedness. By $r_{i,j}$, the most connected were flock 1 and 10 and 4 and 10 ($r_{1,4}$ and $r_{1,10}$ ~ 0.0025). The most connected flock to the rest of the population was flock 1 ($\bar{r_{1}}$ ~ 0.0005), and the least connected one was flock 7 ($\bar{r_{7}}$ = 0). The results indicate poor connectedness in this population, as the benchmark for this statistic ($r_{i,j}$) is 0.05 for “good” and $r_{\left(i,j\right)}\;$ = 0.10 for “superior” connectedness. 

However, the $r_{i,j}$ was uncorrelated to $PEVD_{ind\left(i,j\right)}$, $PEVD_{group\left(i,j\right)}$, and $CD_{ind\left(i,j\right)}$ (Figure 8), making this conclusion unreliable.

## 4. Discussion

The estimation of $N_{e}$ based on inbreeding rate or coancestry rate from genealogical data heavily depends on available ancestral information for the reference population. A thorough quality control of the pedigree in the preliminary analysis was required to construct a valid reference population, since only informative animals with sufficient ancestral information fairly contribute to the estimates of population-specific parameters. The determined steep decline in pedigree completeness with each ancestral generation was not surprising because a systematic recording scheme in this population started about two decades ago, with very scarce prior information. However, the determined low *PCI* and other quality control parameters (*NEG*, *NFG*, and *NMG*) were related to the very old animals in the pedigree, while the younger ones, i.e., those born from 2010 onwards, showed a much better pedigree profile. Therefore, only the latter were included in the analysis of *F* and *Ne*, with additional constraints set at *NEG* ≥ 4 or *NFG* ≥ 3. 

Effective population ($N_{e}$) size has been one of the key parameters in population and quantitative genetics since its conceptualization [32]. By representing the size of an idealized population that would experience the same rate of genetic drift as in the observed population, the $N_{e}$ impacts inbreeding rate and thus the loss of genetic diversity. Such a high inbreeding and low effective population size ($Ne_{NEG\geq 4}=Ne_{NFG\geq 3}=73$) estimated as the mean rate of increase in coancestry [17] were surprising at first sight, but not completely unexpected by taking into account the size of the census population. To be more specific, small populations without a specifically designed mating plan tend to have proportionally more animals with higher *F* than larger populations, due to fewer candidate animals to select from. A decrease of $N_{e}$ in the recent period has been detected, regardless of imposed constraints related to pedigree information. The estimated $Ne_{NFG\geq 3}$ and $\Delta Ne_{NFG\geq 3}$ were very similar to $Ne_{NEG\geq 4}$ and $\Delta Ne_{NEG\geq 4}$, respectively. From our point of view, $Ne_{NFG\geq 3}$ and $\Delta Ne_{NFG\geq 3}$ represent better estimates in this population due to better detection of direct recent inbreeding. However, pruning of the animals solely on the number of fully known generations could sometimes lead to underestimation of the inbreeding load, which led us to retain both obtained results. 

The obtained results pertaining to population-specific parameters such as *F* and $N_{e}$ imply that a specially designed long-term breeding plan should be immediately applied in order to maintain the genetic variability of the breed. On the other hand, selection towards dairy traits should also be applied in order to provide the cost-effective self-sufficiency of the breed. The optimum contribution selection approach (OCS), which balances selection progress and preservation of genetic variability [33], seems the best option as it is the only way to reconcile these two confronting concepts. With the OCS approach, genetic contributions of selection candidates to the next generation are optimized to balance benefits and risks, usually by maximizing genetic gain and restricting mating of closely related selection candidates.

The desire of breeders to use the entire available genetic pool for selection progress in this population depends not only on genetic variability, but also on some other important characteristics of the population such as connectedness between flocks. The impossibility to disentangle genetic from environmental effects in disconnected flocks using the classical pedigree-based BLUP methodology leads to bias in the ranking of animals based on estimated breeding values. This issue has been addressed in many sheep breeding programs where connectedness between management units tends to be low [34,35]. 

Analysis of connectedness was conducted to evaluate the possibility of an unbiased across-flock genetic evaluation system. Four different statistical measures of connectedness were examined. The *PEVD* and *CD* statistics were highly correlated to each other, but uncorrelated to *r.* It is very important to address here that both the *PEVD* and the *CD* statistics, which were considered to be the most accurate measures of connectedness, have no known benchmark for evaluation of connectedness. From this point of view, despite being useful in detecting flocks that tend to share breeding animals more frequently and vice versa, these results (*PEVD* and *CD*) were insufficiently informative to tell us more about bias in ranking of the animals EBVs. Fortunately, this was not the case for the r connectedness statistic. By analyzing connectedness in the simulated sheep data, [20] reported that an $r_{i^{\prime }j^{\prime }}$ of 0.05 corresponds to ~80% reduction in bias and an $r_{i^{\prime }j^{\prime }}$ of 0.10 to ~90% reduction in bias (in comparison to disconnected flocks) and concluded that benchmarks of 0.05 represent “good” connectedness, and benchmarks of 0.10 indicate “superior” connectedness, irrespective of heritability. Therefore, based the obtained $r_{i^{\prime }j^{\prime }}$ it turned out that connectedness in this sheep population has not been at a sufficient level for unbiased ranking of EBVs, which has been caused by the absence of artificial insemination and infrequent exchange of breeding animals between flocks. In order to increase the link between flocks in this population, some of the long-term specially designed breeding schemes need to be implemented. The best-known schemes to provide connections between different management units are the rotation of rams between herds (circle rams) and the sire references scheme [36,37]. 

The results of this study showed that this sheep population is experiencing the unfavorable loss of genetic variability common in small sheep population under selection. Urgent action is required to slow down this process and create the necessary conditions (connectedness) to successfully utilize the entire genetic pool of the breed in a long-term selection. The transition to genomic selection would probably be beneficial for both addressed issues. In terms of maintaining genetic variability, it should provide more accurate information on individual and population genetic parameters. In terms of genetic evaluation and selection, it should compensate for poor connectedness between flocks [25,26].

## 5. Conclusions

Deep pedigree analysis revealed a decreasing rate of effective population size and unfavorable population structure (genetically disconnected flocks) for unbiased across-flock genetic evaluation. The optimum contribution selection appears to be the most appropriate selection strategy for this population to balance selection gain with loss of genetic variability. Structural weaknesses of the population should be improved in order to exploit the entire available genetic pool of the breed in the future and to benefit as much as possible from investments in genomic selection.

## Figures and Tables

**Table 1:** Basic descriptive statistics of the pedigree (*n* = 6866).

	*NEG*	*NFG*	*NMG*	*PCI*
min	0.00	0.00	0.00	0.00
max	6.72	5.00	12.00	1.00
median	2.13	1.00	3.00	0.50
mean	2.15	1.46	3.35	0.47

*NEG*, number of equivalent generations; *NFG*, number of fully traced generations; *NMG*, number of maximum known generations; *PCI*, pedigree completeness index.

**Table 2:** Distribution of the pedigree completeness index (*PCI*).

Lower *PCI*	Upper *PCI*	Frequency	%	Cumulative %
0	0.2	1588	23.19	23.19
0.2	0.4	1496	21.85	45.04
0.4	0.6	1040	15.19	60.23
0.6	0.8	1222	17.85	78.08
0.8	1	1520	22.2	100.00

**Table 3:** Distribution of full known generations (*NFG*).

*NFG*	Frequency	%	Cumulative %
0	1588	23.19	23.19
1	2011	29.37	52.56
2	1963	28.67	81.23
3	1090	15.92	97.15
4	174	2.54	99.69
5	21	0.31	100

*NFG*, number of fully traced generations.

**Table 4:** Distribution of the pedigree completeness index (*PCI*) in the reference population (2010–2018).

Lower *PCI*	Upper *PCI*	Frequency	Cumulative	%	Cumulative %
0	0.2	203	203	9.19	9.19
0.2	0.4	127	330	5.75	14.95
0.4	0.6	123	453	5.57	20.52
0.6	0.8	464	917	21.01	41.53
0.8	1	1309	2226	59.28	100.82

**Table 5:** Distribution of full known generations (*NFG*) in the reference population (2010–2018).

*NFG*	Frequency	%	Cumulative %
0	203	9.19	9.19
1	286	12.95	22.15
2	627	28.4	50.54
3	900	40.76	91.3
4	172	7.79	99.09
5	20	0.91	100

**Table 6:** Distribution of the estimated coefficient of inbreeding (*F*) in the reference population (2010–2018).

		Reference Population		$F_{NEG\geq 4}$		$F_{NFG\geq 3}$	
Lower *F*	Upper *F*	Frequency	%	Frequency	%	Frequency	%
0.00	0.10	1678	76.0	523	61.67	695	63.64
0.11	0.20	327	14.8	188	22.17	253	23.17
0.21	0.30	149	6.8	89	10.5	96	8.79
0.31	0.40	47	2.1	41	4.83	41	3.75
0.41	0.50	7	0.3	7	0.83	7	0.64

**Table 7:** Estimated effective population size and coefficient of inbreeding in recent populations (animals with NEG > 4).

GI (Years)	$Ne_{NEG\geq 4}$	$Nt_{NEG\geq 4}$	$Ne_{NEG\geq 4}$ $/Nt_{NEG\geq 4}$	$F_{NEG\geq 4}$
1 (2012–2015)	92	903	0.10	0.11
2 (2013–2016)	85	768	0.11	0.11
3 (2014–2017)	78	663	0.12	0.10
4 (2015–2018)	73	499	0.15	0.10

**Table 8:** Estimated effective population size in recent populations (animals with NFG > 3).

GI (Years)	$Ne_{NFG\geq 3}$	$Nt_{NFG\geq 3}$	$Ne_{NFG\geq 3}$ $/Nt_{NFG\geq 3}$	$F_{NFG\geq 3}$
1 (2012–2015)	93	897	0.10	0.10
2 (2013–2016)	87	753	0.11	0.10
3 (2014–2017)	79	638	0.12	0.10
4 (2015–2018)	73	489	0.15	0.11

## Data Availability

The data presented in this study are available on request from the corresponding author. The data are not publicly available to preserve privacy of the data.
